# Targeting the Bromodomain of BRG-1/BRM Subunit of the SWI/SNF Complex Increases the Anticancer Activity of Temozolomide in Glioblastoma

**DOI:** 10.3390/ph14090904

**Published:** 2021-09-06

**Authors:** Chuanhe Yang, Yinan Wang, Michelle M. Sims, Yali He, Duane D. Miller, Lawrence M. Pfeffer

**Affiliations:** 1 Department of Pathology and Laboratory Medicine, Center for Cancer Research, College of Medicine, University of Tennessee Health Science Center, Memphis, TN 38163, USA; cyang@uthsc.edu (C.Y.); ywang127@uthsc.edu (Y.W.); msims7@uthsc.edu (M.M.S.); 2 Department of Pharmaceutical Sciences, College of Pharmacy, University of Tennessee Health Science Center, Memphis, TN 38163, USA; yhe33@uthsc.edu (Y.H.); dmiller@uthsc.edu (D.D.M.)

**Keywords:** glioblastoma, BRG1, BRM, SWI/SNF, gene expression, temozolomide, bromodomain inhibitor, small molecule inhibitor, cancer

## Abstract

Glioblastoma (GBM) is a deadly and incurable brain cancer with limited therapeutic options. PFI-3 is a small-molecule bromodomain (BRD) inhibitor of the BRM/BRG1 subunits of the SWI/SNF chromatin remodeling complex. The objective of this study is to determine the efficacy of PFI-3 as a potential GBM therapy. We report that PFI-3 binds to these BRDs when expressed in GBM cells. PFI-3 markedly enhanced the antiproliferative and cell death-inducing effects of temozolomide (TMZ) in TMZ-sensitive GBM cells as well as overcame the chemoresistance of highly TMZ-resistant GBM cells. PFI-3 also altered gene expression in GBM and enhanced the basal and interferon-induced expression of a subset of interferon-responsive genes. Besides the effects of PFI-3 on GBM cells in vitro, we found that PFI-3 markedly potentiated the anticancer effect of TMZ in an intracranial GBM animal model, resulting in a marked increase in survival of animals bearing GBM tumors. Taken together, we identified the BRG1 and BRM subunits of SWI/SNF as novel targets in GBM and revealed the therapeutic potential of applying small molecule inhibitors of SWI/SNF to improve the clinical outcome in GBM using standard-of-care chemotherapy.

## 1. Introduction

Gliomas are the most common primary intracranial neoplasms in adults and a leading cause of cancer-related morbidity and mortality in the United States [1]. While grade I glioma is the least malignant brain tumor, grade IV glioma (GBM, glioblastoma) is the most aggressive and the deadliest brain tumor. Surgical resection of GBM remains the primary treatment modality, combined with adjuvant chemotherapy temozolomide (TMZ) and radiation therapy, which only provide a slight improvement in the disease course and outcome [2]. The overall median time for GBM recurrence after treatment is 7 months, and its 5-year overall prognosis is dismal (<10% survival), which has remained relatively unchanged for decades [1].

More than 20% of human cancers bear mutations in the mammalian ATP-dependent chromatin remodeling of the SWI/SNF complex, which is an evolutionarily conserved multi-subunit complex critical for gene regulation, differentiation, DNA repair, and development [3,4]. The two catalytic subunits of the SWI/SNF complex are BRM (Brahma/*SMARCA2*) and BRG1 (Brahma-related gene 1/*SMARCA4*). In cancers of the lung, ovaries, skin, and blood (lymphoma), BRG1 functions as a tumor suppressor with silencing or loss-of-function mutations being highly enriched [4,5,6,7,8]. However, BRG1 was found to play a pro-tumorigenic role in acute myeloid leukemia by promoting cancer cell survival and facilitating MYC expression [9]. In GBM, BRG1 mutations are relatively rare [10], and we found that BRG1 is highly expressed in GBM tumor tissue and GBM cells grown in vitro [11,12]. We also showed that BRG1 is essential for maintaining the stem cell-like identity of GBM cancer stem cells (GSCs) [11], and BRG1 knockdown in GSCs and in GBM cell lines increases chemosensitivity to the standard-of-care DNA alkylating agent TMZ [11,12].

PFI-3 is a small-molecule inhibitor of the bromodomains (BRDs) of the BRM/BRG1 subunits [13,14]. The BRDs of these subunits read histone acetylation marks, while the ATPase domains propel DNA along the histone surface. While the ATPase domain of these subunits is essential for tumor cell proliferation and has been targeted with small molecular anticancer agents [15,16], the BRD of BRG1 apparently is dispensable for cell proliferation and is far less studied as a drug target [17]. In the present study, we examined the mechanism of action of PFI-3 in GBM cells. We show that PFI-3 binds to the BRDs of both BRM and BRG1, but PFI-3 does not alter GBM cell proliferation. However, PFI-3 increased the sensitivity of MT330 and LN229 GBM cell lines to the DNA alkylating agent TMZ and overcame the chemoresistance of the TMZ-resistant T98G GBM cell line as determined by live cell proliferation and cell death assays. PFI-3 also altered gene expression in GBM cells by enhancing the basal and interferon (IFN)-induced expression of several IFN-responsive genes. Moreover, we show that a combination of TMZ and PFI-3 has marked anticancer activity in the intracranial animal model of GBM by increasing animal survival. Together, our data identify SWI/SNF as a novel target in GBM and reveal the therapeutic potential of applying small molecules to inhibit SWI/SNF function to improve the clinical outcome of GBM.

## 2. Results

### 2.1. BRG1 Is Highly Expressed in GBM Tumor Tissue and in GBM Cells

**Figure 1:**
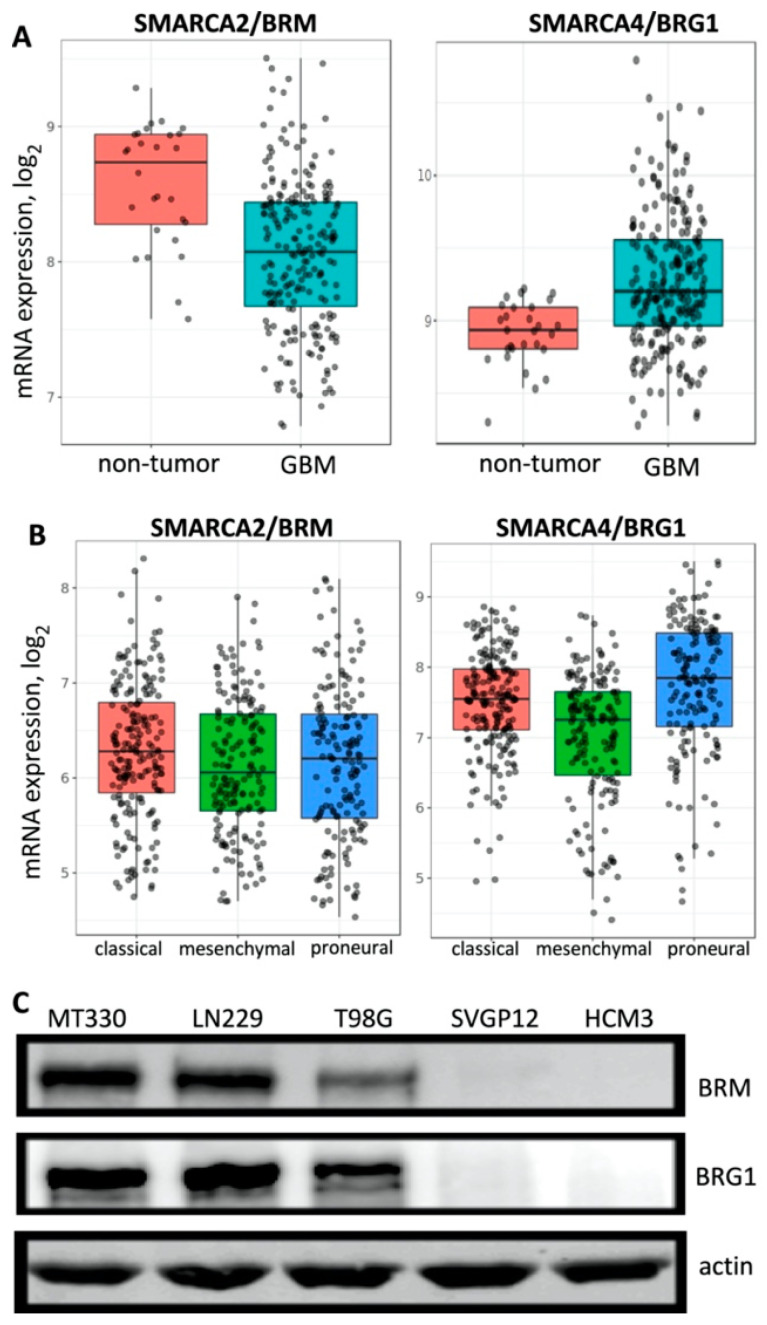
BRG1 and BRM gene and protein expression. (**A**) A total of 28 non-tumor tissue samples and 219 GBM patient samples in the REMBRANDT database were compared for BRG1 and BRM expression. (**B**) BRG1 and BRM expression in the different GBM molecular subtypes in the REMBRANDT database. (**C**) Protein lysates from GBM cell lines (T98G, MT330, and LN229) and normal human glial cells (SVGp12 and HCM3) were blotted for BRG, BRM, and actin.

Mutation and deletion of BRG1/SMARCA4 have been shown to contribute to a range of human malignancies [10,18], suggesting that the SWI/SNF complex acts as a tumor suppressor in these cancers. However, recent evidence showed that the SWI/SNF may play a pro-tumorigenic role in other cancers [9,11,12]. For example, we previously reported in GBM, while EGFR amplification and PTEN deletion arise frequently, the BRG1 and BRM catalytic subunits of SWI/SNF are rarely altered [12]. We first examined the expression of BRM and BRG1 subunits in the REMBRANDT database of normal tissue and GBM tumor samples and found that, while BRG1 expression in GBM was higher than in non-tumor tissue, BRM expression was lower in GBM tumor tissue as compared to non-tumor samples (Figure 1A). Gene expression analysis showed that GBM may be classified into three distinct molecular subtypes: classical, mesenchymal, and proneural [19]. We then determined using the GlioVis data portal if there were differences in expression of BRG1 and BRM in these GBM molecular subtypes. We found that BRG1 and BRM were expressed in all three molecular subtypes of GBM in the REMBRANDT database with only minor variations in the expression of the SWI/SNF catalytic subunits (Figure 1B). We next examined the protein expression of BRG1 and BRM in several human GBM (MT330, LN229, and T98G) cell lines and normal glial cell lines (SVGP12 and HCM3). Whole-cell protein lysates were prepared and examined by immunoblotting. We found that both BRG1 and BRM were expressed in the three established GBM (MT330, LN229, and T98G) cell lines tested, but neither catalytic subunit was detected in normal human astrocytes (SVGp12) and microglial (HCM3) cells (Figure 1C).

### 2.2. PFI-3 Binds to the Bromodomains of BRG1 and BRM

**Figure 2:**
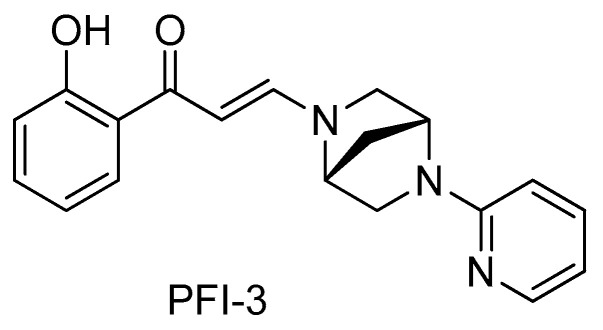
The chemical structure of PFI-3.

Both BRG1 and BRM contain BRDs, which are evolutionarily conserved protein-protein interaction modules present in ~50 proteins in humans that bind acetyl-lysine on protein and histone tails [20,21]. The small molecule BRD inhibitor PFI-3 (shown in Figure 2) was identified by profiling against 48 BRDs and shown to have high selectivity for the BRDs of BRG1and BRM [13].

**Figure 3:**
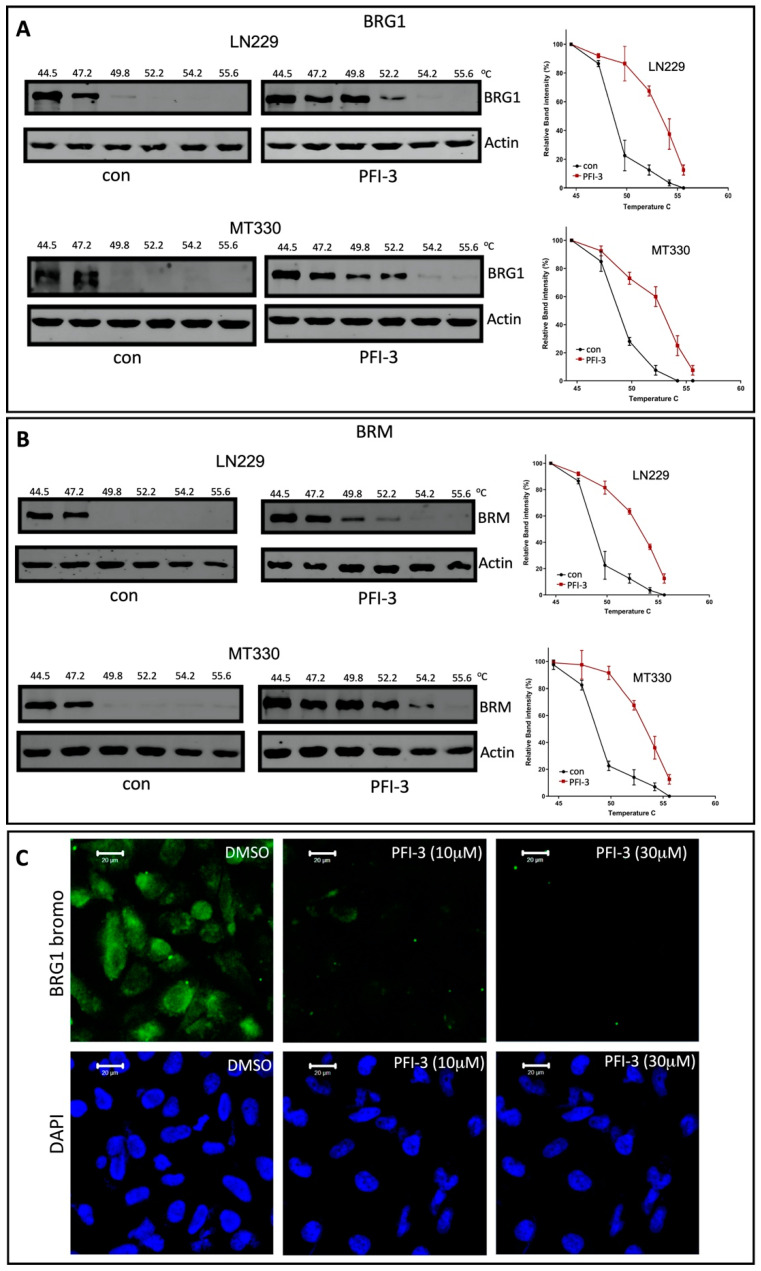
Binding assays for PFI-3 to BRG1 and BRM BRDs. (**A**,**B**) For the cellular thermal shift assay, MT330 cells expressing the BRG1 (**A**) and BRM (**B**) epitope-tagged bromodomain construct were treated with PFI-3 (30 mM) or DMSO for 2 h. After heating over a temperature range from 44.5 to 55.6 °C for 5 min, the cells were lysed, placed on ice at 4 °C, and then immunoblotted for BRG1 or actin. (**C**) MT330 cells expressing a GFP-tagged BRG1 bromodomain construct containing a nuclear localization signal were treated PFI-3 (10 or 30 μM), or DMSO. After 2 h, the cells were fixed, DAPI counterstained, and examined by confocal microscopy.

To characterize the BRD-binding activity of the small molecule BRD inhibitor PFI-3 to these SWI/SNF subunits, we developed a cellular thermal shift assay (CETSA) [22] with MT330 and LN229 GBM cells transduced with either an epitope-tagged BRG1 or BRM BRD. A CETSA measures thermostability and the epitope-tagged BRD becomes more thermostable when an inhibitory molecule was bound. In brief, MT330 cells expressing the BRG1 BRD construct were treated with PFI-3 or DMSO for 2 h. After heating over a temperature range from 44.5 to 55.6 °C for 5 min, the cells were lysed and then immunoblotted for BRG1. As shown in Figure 3A, PFI-3 increased the thermostability of the BRG1 BRD expressed in both MT330 and LN229 cells, with the protein band being undetectable at 54.2 °C in PFI-3 treated cells versus 49.8 °C in the vehicle control. A similar finding was obtained by CETSA when an epitope-tagged BRM BRD was expressed in both MT330 and LN229 GBM cells. As shown in Figure 3B, the BRD became more thermostable when PFI-3 was bound to it as compared to the vehicle control. As a complementary approach, we next developed a chromatin-based assay for BRD binding [17]. A GFP-tagged BRG1 BRD containing a nuclear localization signal was expressed in MT330 GBM cells. Since the BRG1 construct contained a nuclear localization signal, the GFP fluorescence localizes to the nucleus and persists there as the BRG1 BRD became bound to chromatin. PFI-3 displaced BRG1 and BRD from the nucleus in a dose-dependent manner (Figure 3C). Taken together, these results demonstrate that PFI-3 binds to BRDs of both catalytic subunits of SWI/SNF.

### 2.3. PFI-3 Treatment Increases TMZ-Sensitivity of GBM Cells

**Figure 4:**
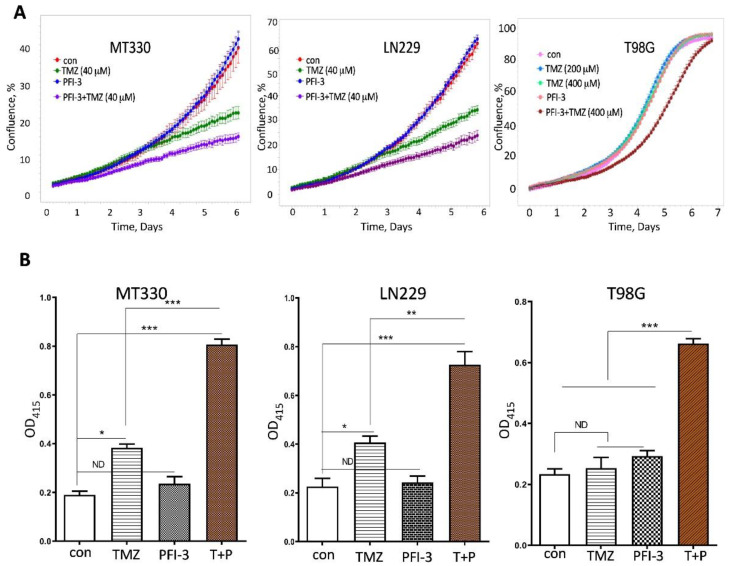
PFI-3 sensitizes GBM cells to TMZ. (**A**) MT330, LN229, and T98G GBM cells were treated with TMZ as indicated, and PFI-3 (2 μM), and cell proliferation was determined by Incucyte live cell analysis. The result of one of two individual experiments performed in triplicate is shown. (**B**) MT330, LN229, and T98G GBM cells were treated with TMZ (50, 50, and 500 μM, respectively) and PFI-3 (2 μM), and cell death was determined by a cell death ELISA. *p* values < 0.05 (*), 0.01 (**) and 0.001 (***).

We have previously shown that knockout of BRG1 in GBM cell lines increased their sensitivity to TMZ [12]. The genetic dependency on SWI/SNF led us next to whether the small molecule dual BRG1/BRM BRD inhibitor, PFI-3, was also able to sensitize GBM cells to TMZ. We previously found that MT330 and LN229 cells are highly sensitive to low doses of TMZ [12]. Thus, we treated these GBM cells with PFI-3 in the presence or absence of 40 mM TMZ, and measured GBM proliferation by live cell analysis. PFI-3 alone had no effect on the proliferation of either MT330 or LN229 GBM cells, which is consistent with the findings that PFI-3 did not exhibit toxicity against any of the NCI-60 panel of tumor cell lines [13]. Most importantly, PFI-3 treatment increased the sensitivity of both MT330 and LN229 GBM cells to the antiproliferative effect of TMZ (Figure 4A). Furthermore, T98G GBM cells are highly TMZ-resistant with no effect on cell proliferation observed at as high a TMZ concentration of 400 mM, which is a 10-fold higher than the dose used in MT330 and LN229 cells in our experiments (Figure 4), Nonetheless, PFI-3 treatment also sensitized these highly TMZ-resistant cells to the antiproliferative effect of TMZ.

PFI-3 had a similar chemosensitizing effect when TMZ-induced cell death in GBM cells was determined by sensitive ELISA assays (Figure 4B). While TMZ alone induced cell death in both MT330 and LN229 cells, TMZ alone had no effect on cell death in T98G cells. In addition, PFI-3 alone did not induce cell death in any of these GBM cells. However, PFI-3 treatment markedly increased the sensitivity of all GBM cells to TMZ-induced cell death, surprisingly even in the TMZ-resistant T98G GBM cells. Our results demonstrate that PFI-3 sensitizes not only TMZ-sensitive but also TMZ-resistant GBM cells to the inhibitory effect of TMZ on cell proliferation as well as TMZ-induced cell death by TMZ in vitro.

### 2.4. PFI-3 Regulates IFN-Induced Gene Expression in GBM Cells

**Figure 5:**
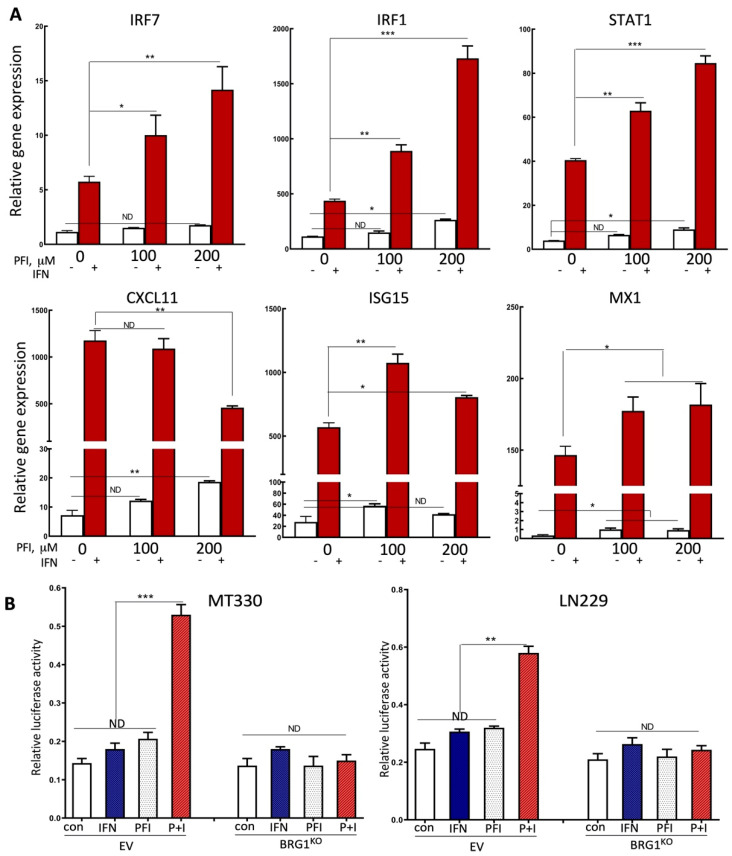
The effects of PFI-3 on IFN-induced gene expression in GBM cells. (**A**) RNA was prepared from MT330 cells treated for 6 h with PFI-3 (concentrations indicated) and IFN (1000 IU/mL), and the expression of the indicated genes was determined by qPCR and normalized to actin expression. (**B**) MT330 and LN229 BRG1^KO^ cells were transiently co-transfected with IRF-1 luciferase gene reporter construct and Renilla construct, and 24 h after transfection treated with PFI-3 (10 μM)) or IFN (1000 IU/mL) alone or in combination, and then harvested to measure reporter activity. Luciferase activity was normalized to Renilla activity. *p* values < 0.05 (*), 0.01 (**) and 0.001 (***).

The SWI/SNF chromatin remodeling complex plays a critical role in regulating gene transcription [23,24]. In a previous study, we found that knockdown of BRG1 in GSCs selectively regulated the expression of specific genes, in particular genes in the IFN response pathway, including several well-known IFN-stimulated genes (ISGs) [11]. To investigate the mechanisms that may underlie the activity of the small molecule BRD inhibitor PFI-3 in GBM cells, we examined the effect of PFI-3 on the expression of ISGs in GBM cells. We extracted RNA from control and PFI-3-treated MT330 cells in the presence and absence of human IFN, and then determined the expression of the following genes by quantitative real-time PCR using gene-specific primers for STAT1, CXCL11, MX1, ISG15, IRF1, and IRF7. As expected, all these genes are highly responsive to IFN, being induced by at least 10-fold (Figure 5A). Interestingly, PFI-3 increased the IFN-induced expression in a subset of these genes, including IRF1, IRF7, and STAT1, while having little effect on ISGs such as ISG15 and MX1, and a slightly inhibitory effect on CXCL11 at the highest does of PFI-3 used. Furthermore, PFI-3 also slightly increased the basal expression of all ISGs in a dose-dependent manner.

Since IRF1 was among the ISGs that were highly responsive to PFI-3 treatment (Figure 5A), we next determined whether the effect of PFI-3 was directly on IRF-1 gene transcription. In brief, MT330 and LN229 GBM cells were transiently transfected with the IRF-1-dependent luciferase reporter construct, and at 72 h after transfection, cells were treated with PFI-3 alone or in the presence of IFN. In both MT330 and LN229 cells, the combination of PFI-3 and IFN robustly induced IRF-1 dependent promoter expression. We next determined the effect of PFI-3 on IRF-1 gene transcription in MT330 and LN229 BRG1^KO^ GBM cells, in which the BRG1 subunit of SWI/SNF was knocked out in each cell line by CRISPR/Cas9 gene-editing [12]. Transient transfection of the IRF-1 dependent reporter construct in these BRG1^KO^ cells nearly completely ablated the combined effect of PFI-3 and IFN as compared to parental wild-type GBM cells (Figure 5B), which strongly suggested that the effect of PFI-3 was highly dependent on the BRG1 subunit of SWI/SNF.

### 2.5. PFI-3 Enhances the Anticancer Activity of TMZ on GBM Tumorigenesis

**Figure 6:**
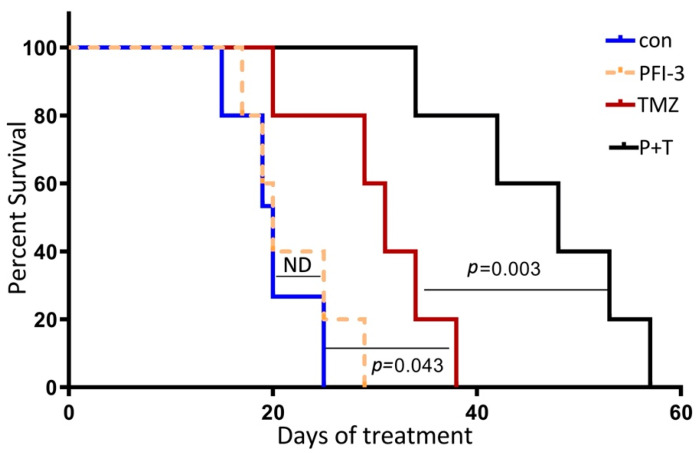
PFI-3 enhances the anticancer activity of TMZ on GBM tumorigenesis. Luciferase-expressing MT330 cells were injected into the brains of 5-week-old female immunocompromised NSG mice. After tumors were identified by BLI (~10 days after tumor cell injection), mice intraperitoneally injected thrice weekly with TMZ (60 mg/Kg body weight) or PFI-3 (10 mg/Kg body weight) alone or in combination. Kaplan–Meier analysis of the survival data (8 mice/group) was performed.

Based on our findings that PFI-3 enhanced the antiproliferative effects of TMZ in vitro, we next examined whether PFI-3 enhanced the anticancer activity of TMZ in a highly relevant animal model of GBM. To assess the biological activity of PFI-3 in the orthotopic environment of the brain, luciferase-expressing MT330 GBM cells were injected into the brains of immunocompromised NSG mice. Once brain tumors were identified at ~10 days after tumor cell injection by bioluminescent imaging (BLI), mice were subjected to thrice weekly intraperitoneal injections of TMZ or PFI-3 alone or in combination. The mean survival of control mice was ~20 days after tumor cell injection (Figure 6). While PFI-3 treatment alone had no effect of mouse survival, TMZ alone enhanced mouse survival (mean survival of 32 days). Most importantly, the combination of TMZ with PFI-3 markedly enhanced animal survival (mean survival of 50 days) as compared to the effect of TMZ alone. While the blood–brain barrier (BBB) is a critical barrier to the clinical efficacy of therapeutics to treat GBM, we provide compelling evidence that PFI-3 has anticancer activity in intracranial GBM xenografts when combined with standard-of-care TMZ therapy and crosses the BBB.

## 3. Discussion

The SWI/SNF chromatin remodeling complex plays critical roles in diverse biological processes, such as cell proliferation, differentiation, metabolism, and DNA repair, by binding to the promoters and enhancers of specific genes to regulate gene expression [25,26,27,28,29,30]. Genomic alterations of the BRG1and BRM subunits of SWI/SNF contribute to a range of human malignancies, indicating that they may function as tumor suppressors [10,18]. However, recent studies suggest that in some cancers, BRG1/BRM may play a pro-tumorigenic role [9,11,12]. In the present study, we found that BRG1 expression was higher in GBM tumor tissue compared to non-tumor tissue, while BRM was expressed at lower levels in GBM compared to non-tumor tissue. Both subunits were expressed in all three molecular GBM subtypes, but there was generally a lower expression of BRM as compared to BRG1. Taken together, these findings suggest that BRG1 and not BRM plays a pro-tumorigenic role in GBM, and that BRG1 may be an attractive therapeutic target in GBM. 

The BRG1 and BRM catalytic subunits of SWI/SNF have two important functional domains: a BRD that reads histone acetylation and an ATPase domain that propels DNA along the histone surface. The ATPase domain of these subunits is essential for tumor cell proliferation and thus has been a target of small molecule anticancer agents in several cancer types [15,16]. However, although the BRD has been a target of drug evaluation in some biological studies, BRD has not been thoroughly investigated as a drug target in cancer. PFI-3, a small-molecule inhibitor of the BRM/BRG1 BRD, was found to enhance the differentiation of embryonic stem cells and adipocytes but does not alter their cell proliferation [13,14]. In GBM, we previously found that PFI-3 enhances the sensitivity of GSCs to DNA alkylating agents but does not have a significant effect on stem cell proliferation. In GBM, while GSCs play an important role in tumor recurrence and resistance to chemotherapy, a large fraction of the tumor is comprised of differentiated GBM tumor cells, which also play an important role in GBM tumorigenesis. We found that while both BRG1 and BRM are expressed in a variety of GBM cell lines, neither subunit of SWI/SNF is expressed in normal human microglial cells.

The small molecule BRD inhibitor PFI-3 was identified by profiling against 48 BRDs and found to be selectively bound to the BRDs of BRG1and BRM [13]. To characterize the BRD binding activity of PFI-3 to BRG1 and BRM in GBM cells, we developed a CETSA using MT330 and LN229 GBM cells transduced with either an epitope-tagged BRG1 or BRM BRD and found that PFI-3 bound well to both BRDs. The binding of PFI-3 to BRG1 was also confirmed by using a fluorescent chromatin-based assay. Our results are consistent with the previous finding that PFI-3 is directly bound to the BRG1 and BRM BRDs in HeLa cells [17].

We then found that PFI-3 sensitized GBM cells to the effects of anticancer effects of TMZ on cell proliferation and death. Interestingly, sensitization to TMZ was also observed in a GBM cell line highly resistant to TMZ. It is important that in all GBM cell lines examined that PFI-3 alone had no effect on cell proliferation or death, indicating that these effects are not due to general toxicity of PFI-3. These data are consistent with PFI-3 not being toxic against any of the NCI-60 panel of tumor cell lines [13]. Moreover, we have previously shown that knockout of BRG1 in MT330 and LN229 GBM cell lines increased their sensitivity to TMZ [12] as well as inhibited cell proliferation [12]. Taken together, our results indicate that PFI-3 phenocopied the TMZ sensitization by genetic ablation of BRG1. However, the antiproliferative action of BRG1-KO in GBM cells that may reflect the function of the ATPase domain of BRG1. Consistent with this function of the ATPase domain, recent studies in uveal melanoma show that small molecule inhibitors of the BRG1/BRM ATPase domains have antiproliferative activity [16].

Based on our previous finding, knockdown of BRG1 subunit of SWI/SNF in GSCs selectively regulated the expression of several well-known ISGs [11]. We examined the effect PFI-3 on the expression of ISGs in GBM cells. Interestingly, PFI-3 increased the IFN-induced expression of a subset of these genes, including IRF1, IRF7, and STAT1, but not of other ISGs such as ISG15, MX1, and CXCL11. Furthermore, PFI-3 alone also slightly increased the basal expression of these ISGs in a dose-dependent manner. IRF1, IRF7, and STAT1 are genes that play critical roles not only in the anticancer activity of IFN but also in innate immunity. We also found that that PFI-3 directly regulated IRF-1 gene transcription. Intriguingly, BRG1-KO ablated the effect of PFI-3 on IRF-1 gene transcription, which is consistent with PFI-3 targeting the BRG1 BRD.

Finally, we showed that PFI-3 enhanced the anticancer activity of TMZ in an orthotopic animal model of GBM by injecting GBM cells into the brains of immunocompromised mice. Once tumors were identified, mice were treated by intraperitoneal injection of TMZ or PFI-3 alone or in these two drugs in combination. We found that the combination of TMZ with PFI-3 markedly enhanced animal survival as compared to the effect of TMZ alone, while PFI-3 had no effect on animal survival. Since the blood–brain barrier is a critical impediment to the clinical efficacy of GBM therapeutics, we found that PFI-3 has anticancer activity in intracranial GBM xenografts when combined with standard-of-care TMZ therapy and crosses the BBB. Consistent with the present findings, in previous studies we showed that BRG1 knockdown in GSCs resulted in the enhanced sensitivity of mouse tumors to TMZ [11]. Thus, PFI-3 once again phenocopied the effect of genetic ablation or knockdown of BRG1. However, in GSCs BRG1-knockdown increases GBM tumor growth, but the BRD inhibitor alone does not alone effect tumorigenesis. Once again, it is important to note that, while small molecule BRG1/BRM ATPase inhibitors are antiproliferative in cancer [16], PFI-3 has no direct effect on cancer cell proliferation or death. Taken together, our data identify the BRG1 BRD of SWI/SNF as a novel target in GBM and reveal the therapeutic potential of PFI-3 and other small molecules to inhibit SWI/SNF function to improve the clinical outcome of GBM.

## 4. Materials and Methods

### 4.1. Biological and Chemical Reagents, and Cell Culture

Antibodies to the following proteins were used: BRG1 and BRM (Cell Signaling, Danvers, MA, USA); actin (Santa Cruz Biotechnology, Santa Cruz, CA, USA). PFI-3 was synthesized by methods previously described [31], and TMZ was purchased from Sigma. Chemicals were dissolved in DMSO prior to use and stored in aliquots at −20 °C. Human consensus type I IFN was generously provided by AMGEN. U87 and LN229, obtained from American Type Culture Collection (Manassas, VA, USA), and MT330 GBM [32] cells were grown in DMEM containing 10% fetal bovine serum (Hyclone, Logan, UT, USA) supplemented with penicillin (100 IU/mL) and streptomycin (100 μg/mL) at 37 °C with 5% CO_2_.

### 4.2. Repository of Molecular Brain Neoplasia Data (REMBRANDT) Query

Microarray data from REMBRANDT for all non-tumors (28 samples) and GBM tumors (219 samples) were queried using the GlioVis data portal for visualization and analysis of brain tumor expression datasets [33]. The dataset was filtered for samples having expression data for BRG1 and BRM and the accompanying clinical data. Statistical analyses were performed using Graphpad Prism.

### 4.3. Immunoblot Analysis

Total cell lysates (25 μg) were separated by SDS-PAGE, immunoblotted with antibodies against BRG1, BRM, and actin, and visualized, as previously described [34].

### 4.4. Analysis of Binding to the Bromodomains of BRG1 and BRM

The BRD-binding activity of the small molecule PFI-3 to the BRD of the catalytic subunits of SWI/SNF was assessed by an adaption of a previously described CETSA procedure [22]. MT330 and LN229 GBM cells were transduced with a lentivirus encoding either an epitope-tagged BRG1 or BRM BRD. GBM cells expressing the BRG1 BRD construct were treated with PFI-3 (30 mM) or DMSO as vehicle control for 2 h. After heating over a temperature range from 44.5 to 55.6 °C for 5 min, the cells were lysed, placed on ice, and then immunoblotted for BRG1 or actin. As a complementary approach, we next developed a chromatin-based assay for BRD binding [17]. A GFP-tagged BRG1 BRD containing a nuclear localization signal was expressed in MT330 GBM cells by lentiviral transduction. After transduction, stable pools of cells were treated PFI-3 (10 or 30 μM), or DMSO as a vehicle control. After 2 h, the cells were fixed, DAPI counterstained, and examined by confocal microscopy (Zeiss model LSM700).

### 4.5. Cell Proliferation and Cell Death ELISA Assays

For cell proliferation analysis, cells were plated into 96 well plates (1 × 10^4^ cells/well) and 24 h later incubated into the Incucyte live cell analysis system for up to 7 days in a 37 °C incubator. Cell death detection (ELISA^PLUS^ from Roche, Basel, Switzerland) in 96 well plates was determined according to the manufacturer’s instructions.

### 4.6. Gene Expression Analysis

Gene expression was determined in RNA isolated from human GBM cells as previously described [30]. In brief, total RNA was extracted using the RNeasy mini kits (Qiagen Inc., Frederick, MD, USA). Quantitative real-time PCR (qPCR) was performed using gene-specific primers for STAT1 (forward 5′-CAGCTTGACTCAAAATTCCTGGA-3′ and reverse 5′-TGAAGATTACGCTTGCTTTTCCT-3′), CXCL11 (forward 5′-GACGCTGTCTTTGCATAGGC-3′ and reverse 5′-GGATTTAGGCATCGTTGTCCTTT-3′), MX1 (forward 5′-TCCCACCCTCTATTACTGAATGG-3′ and reverse 5′-GGGAAGGGCAACTCCTGAC-3′), ISG15 (forward 5′-TCCTGGTGAGGAATAACAAGGG-3′ and reverse 5′-GTCAGCCAGAACAGGTCGTC-3′), IRF1 (forward 5′-CTGTGCGAGTGTACCGGATG-3′ and reverse 5′-ATCCCCACATGACTTCCTCTT-3′), IRF7 (forward 5′-GCTGGACGTGACCATCATGTA-3′ and reverse 5′-GGGCCGTATAGGAACGTGC-3′), and BETA-ACTIN (forward 5′-GGACTTCGAGCAAGAGATGG-3′ and reverse 5′-AGCACTGTGTTGGCGTACAG-3′) with an iScript one-step RT-PCR kit containing SYBR Green (Bio-Rad, Hercules, CA, USA). The reaction parameters were as follows: cDNA synthesis at 50 °C for 20 min, transcriptase inactivation at 95 °C for 5 min, and PCR cycling at 95 °C for 10 s and 60 °C for 30 s for 40 cycles. Gene expression was normalized relative to ACTIN expression.

### 4.7. IRF-1-Dependent Luciferase Reporter Assays

To investigate the effect of PFI-3 on IRF-1 transactivation, we transiently transfected LN229 and MT330 GBM cells with the IRF-1 promoter linked to the luciferase reporter gene [35]. At 24 h after transfection, GBM cells were treated with PFI-3 (5 μM) overnight in the presence of IFN (1000 IU/mL, 6 h). Reporter assays were performed as previously described [34].

### 4.8. Tumor Xenograft

Animal experiments were performed in accordance with a protocol approved by the Institutional Animal Care and Use Committee of the University of Tennessee Health Science Center (UTHSC Protocol 17-098, approved on 19 December 2020). Xenografts were established in 5-week-old female NOD.Cg-*Prkdc^scid^* *Il2rg^tm1Wjl^*/SzJ (NSG) mice (Jackson Laboratory, Bar Harbor, ME, USA). Luciferase-expressing MT330 cells (10^6^) were injected stereotactically into the superficial brain parenchyma of NSG mice through a burr hole in the skull as previously described [36]. NSG mice were injected with D-luciferin, and live animal imaging was performed weekly to quantify bioluminescence (BLI) [36,37]. Once tumors were identified by BLI (~10 days after tumor cell injection), mice (~25 gm) were subjected to intraperitoneal injection of TMZ (60 mg/Kg body weight) or PFI-3 (10 mg/Kg body weight) alone or TMZ and PFI-3 together.

### 4.9. Statistical Analyses

At least 2 independent experiments were performed in duplicate, and data were presented as means ± S.D. One-way analysis of variance (One-way ANOVA) and post-hoc least significant difference analysis or Student’s *t*-tests were performed. *p* values < 0.05 (*), 0.01 (**) and 0.001 (***) were considered statistically significant.

## 5. Conclusions

**Figure 7:**
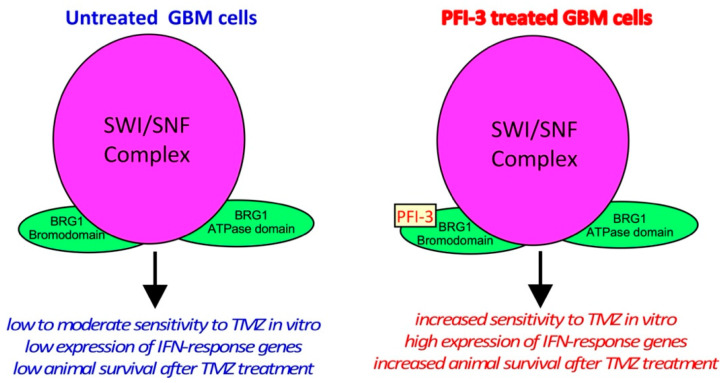
Schematic representation of conclusions.

The small-molecule inhibitor, PFI-3, binds to the BRDs of the BRM/BRG1 subunits of the SWI/SNF complex in GBM cells (Figure 7). PFI-3 treatment of GBM cells markedly enhanced the antiproliferative and cell death-inducing effects of TMZ. PFI-3 slectively altered gene expression in GBM cells and enhanced the basal and interferon-induced expression of a subset of interferon-responsive genes. PFI-3 potentiated the anticancer effect of TMZ in an intracranial animal model of GBM. These studies reveal the therapeutic potential of applying a small molecule inhibitor (PFI-3) of the BRG1 and BRM subunits of SWI/SNF to improve the clinical outcome in GBM using standard-of-care chemotherapy.

## Data Availability

Data is contained within the article.
